# Droplet Microfluidics for Tumor Drug‐Related Studies and Programmable Artificial Cells

**DOI:** 10.1002/gch2.202000123

**Published:** 2021-05-07

**Authors:** Pantelitsa Dimitriou, Jin Li, Giusy Tornillo, Thomas McCloy, David Barrow

**Affiliations:** ^1^ Applied Microfluidic Laboratory School of Engineering Cardiff University Cardiff CF24 3AA UK; ^2^ Hadyn Ellis Building Cardiff University Maindy Road Cardiff CF24 4HQ UK

**Keywords:** artificial cells, droplet microfluidics, drug screening, tumor spheroids

## Abstract

Anticancer drug development is a crucial step toward cancer treatment, that requires realistic predictions of malignant tissue development and sophisticated drug delivery. Tumors often acquire drug resistance and drug efficacy, hence cannot be accurately predicted in 2D tumor cell cultures. On the other hand, 3D cultures, including multicellular tumor spheroids (MCTSs), mimic the in vivo cellular arrangement and provide robust platforms for drug testing when grown in hydrogels with characteristics similar to the living body. Microparticles and liposomes are considered smart drug delivery vehicles, are able to target cancerous tissue, and can release entrapped drugs on demand. Microfluidics serve as a high‐throughput tool for reproducible, flexible, and automated production of droplet‐based microscale constructs, tailored to the desired final application. In this review, it is described how natural hydrogels in combination with droplet microfluidics can generate MCTSs, and the use of microfluidics to produce tumor targeting microparticles and liposomes. One of the highlights of the review documents the use of the bottom‐up construction methodologies of synthetic biology for the formation of artificial cellular assemblies, which may additionally incorporate both target cancer cells and prospective drug candidates, as an integrated “droplet incubator” drug assay platform.

## Graphical abstract

This review summarizes the recent progress of droplet technologies in anticancer drug development. Microfluidic‐formed droplets are utilized in both tumor spheroid formation and smart drug delivery. This work also highlights the current trend of using artificial cells materials for drug metabolism studies. Finally, a novel ‘droplet incubator’ concept as a prospective anticancer drug screening platform is introduced.

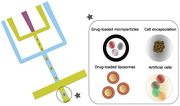

## 1 Introduction

The discovery and pre‐clinical development of anticancer agents often starts with in vitro drug testing before progressing to animal studies, to determine, in vivo, the efficacy and safety of promising therapeutic candidates.^[^ ^1^ ^]^ Over the past decades, advanced methods for the generation of large chemical libraries (i.e., combinatorial chemistry), coupled to affordable laboratory robotics, have promoted the use of in vitro tumor models in high‐throughput drug screenings.^[^ ^2^, ^3^ ^]^ High‐throughput screens for anticancer drugs have been, for a long time, limited to 2D culture of tumor cells, grown as a monolayer on the bottom of a well of a microtiter plate. Compared to 2D cell cultures, 3D culture systems can more faithfully model cell‐cell interactions, matrix deposition and tumor microenvironments, including more physiological flow conditions, oxygen and nutrient gradients.^[^ ^4^ ^]^ Therefore, 3D cultures have recently begun to be incorporated into high‐throughput drug screenings, with the aim of better predicting drug efficacy and improving the prioritization of candidate drugs for further in vivo testing in animals.

Because of the relatively simple, reproducible, amenable to automation and scalable culture methods, single‐cell type and mixed‐cell tumor spheroids, known as multicellular tumor spheroids (MCTSs), are used as 3D models.^[^ ^5^ ^]^ There exists a broad range of natural hydrogels that are compatible with microfluidics, and which provide cancer cells with mechanical cues and adhesion sites to proliferate and grow into MCTSs.^[^ ^6^ ^]^ With the aid of microfluidics and development of more complex 3D tumor models,^[^ ^7^, ^8^ ^]^ and the large‐scale production of tumor spheroids in hydrogels, the number of compounds that could progress to in vivo testing could be restricted, thus reducing the number of animals needed for preclinical studies.

Tumor‐targeted drug delivery using microparticles and liposomes is beneficial compared to conventional drug administration. This is because encapsulated drug dosages can be controlled, healthy tissues can remain unharmed during treatment and drug resistance of cancer cells may be reduced/prevented.^[^ ^9^, ^10^ ^]^ Microparticles and liposomes can be tailored to specifically target tumor sites using molecular conjugates, while avoiding toxic effects.^[^ ^9^ ^]^

**Figure 1:**
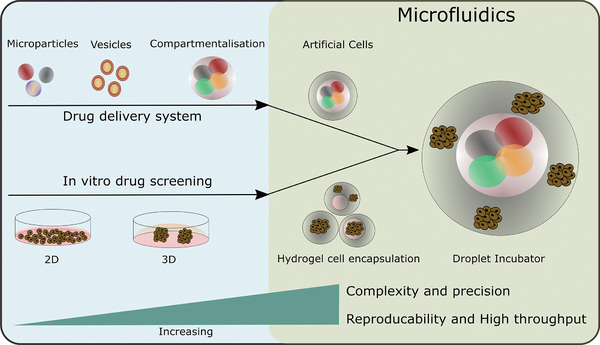
A schematic diagram describing how in vitro drug screening and delivery systems can be utilised as constituents toward novel microfluidic generated drug screening platforms, termed as “droplet incubators”. Multicellular tumor spheroids (MCTSs) in natural hydrogels as in vitro 3D models.

This review discusses the application of droplet‐based microfluidic technologies for the development of accurate in vitro tumor models and improved cancer treatment strategies. The first part of the review centers around the generation of MCTSs in natural hydrogels for a better recapitulation of the in vivo tumor microenvironment. The second section is focused on microparticle and liposomal production for tumor‐targeted drug delivery. Emphases is given to microfluidic methodologies for the production of these systems, and the potential of compartmentalized artificial cells as anticancer drug screening platforms. Finally, the future perspectives of droplet‐based technologies using microfluidics for drug related studies are discussed, where artificial cells, constructed from lipid‐bound inner compartments, and hydrogels, enable a degree of structural rigidity and the additional incorporation of cancer cells and prospective drug candidates, as integrated and interactive assemblies (**Figure** **1**).

MCTSs in the presence of extra‐cellular matrix (ECM) components offer reliable tumor models for drug screening applications.^[^ ^11^ ^]^ These components may be in the form of hydrogels and can recapitulate tumor complexity and multicellular drug resistance.^[^ ^12^ ^]^ Natural hydrogels include natural polymers and ECM components (e.g., polysaccharides, proteins, glycosaminoglycans) derived from living plants or animals, whereas synthetic hydrogels involve synthetic polymers manufactured by chemical methods. In the following subsections of this review, focus will be given to natural hydrogels that provide structural support and cell adhesion sites, in order to assist MCTS growth, by recapitulating the in vivo tumor microenvironments.

### 1.1 Protein, Polysaccharide, and Hybrid Hydrogels

Natural polymers originate from natural sources, often rendering them highly biocompatible, with gelation conditions that can be dependent upon ionic interactions, pH and temperature.^[^ ^13^ ^]^ The majority of natural polymers derived from the ECM of tissues and organs, includes proteins. Matrigel is a basement membrane extracellular (BME) matrix protein extract, which can be derived from Engelbreth‐Holm‐Swarm (EHS) mouse sarcoma.^[^ ^14^, ^15^ ^]^ Collagen Type I is one of the major proteins present in the ECM of tissues and improves cellular attachment in MCTSs studies.^[^ ^6^, ^16^ ^]^ Fibrin and silk fibroin have also been used as hydrogels for tumor related drug studies.^[^ ^17^, ^18^, ^19^, ^20^ ^]^

A wide range of other natural polymers, such as polysaccharides, are used as hydrogels to replicate the structural characteristics of in vivo tissues. Polysaccharide hydrogels, including alginate, chitosan,^[^ ^21^ ^]^ hyaluronic acid,^[^ ^22^ ^]^ and agarose cover a range of materials that have a demonstrated ability to promote cancer cell growth and MCTS formation.^[^ ^23^ ^]^ Alginate is a polyanionic polymer and can be extracted from brown seaweed.^[^ ^24^, ^25^ ^]^ Chitosan is a cationic polyelectrolyte, derived from chitin found in the shells of prawns, lobster, shrimp and grabs, by a process known as deacetylation.^[^ ^26^, ^27^ ^]^ The opposite molecular charges of chitosan and alginate permit them to form polyelectrolyte complexes.^[^ ^28^ ^]^ Literature has reported the application of similar polyelectrolyte complexes for the study of cancer cells,^[^ ^29^ ^]^ multicellular tumor growth formation,^[^ ^30^ ^]^ as well as drug encapsulation and delivery.^[^ ^31^ ^]^

**Figure 2:**
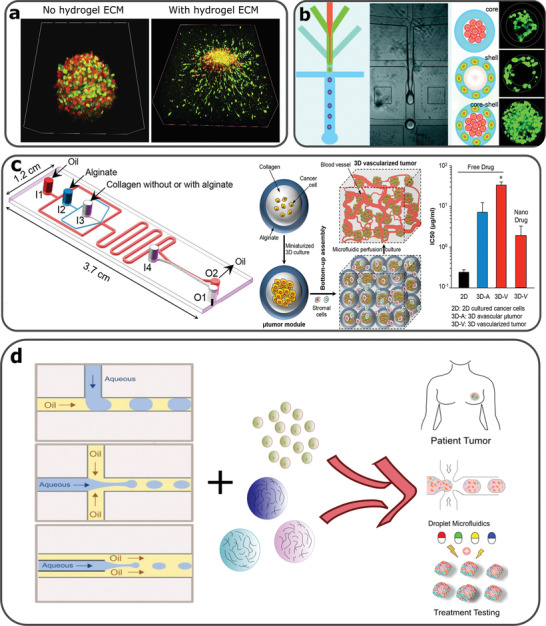
a) Fluorescence microscopy images of MCTS co‐cultures of fibroblasts (green) and cancer cells (red). Tumor invasion occurs only by MCTSs surrounded by the collagen‐alginate 3D hydrogel. Reproduced with permission.^[^ ^33^ ^]^ Copyright 2018, Elsevier. b) Microfluidic assisted formation of liver‐in‐a‐droplet. Microfluidic production of liver‐in‐a‐droplet, where hepatocytes and fibroblasts were encapsulated in the core and shell of the capsule, respectively. Reproduced with permission.^[^ ^62^ ^]^ Copyright 2016, Royal Society of Chemistry. c) A microfluidics device used for production of collagen core and alginate shell capsules used for 3D tumor vascularization experiments. 3D vascularized tumors expressed increased drug resistance in the presence of a commonly used chemotherapeutic drug, although this resistance reduced when treated with drug carrying nanoparticles. Reproduced with permission.^[^ ^63^ ^]^ Copyright 2017, American Chemical Society. d) The rationale of combining microfluidics, hydrogels and cancer cell encapsulation in order to produce high throughput personalized drug treatments. Reproduced with permission.^[^ ^46^ ^]^ Copyright 2019, John Wiley and Sons.^[^ ^64^ ^]^ Copyright 2020, American Chemical Society.

The current interest in hydrogel composites as a platform for MCTS formation and drug testing is increasing.^[^ ^32^, ^33^ ^]^ Collagen and collagen derivatives, such as gelatin, have been tested in combination with polysaccharides, to manufacture in vitro 3D tumor spheroids (**Figure** **2**a).^[^ ^34^ ^]^ MCTS formation has been shown to be dependent on the ratio of protein to polysaccharide scaffold formulation.^[^ ^33^, ^35^ ^]^ Various articles have reported glioblastoma in vitro tumor formation using combination of polysaccharides that mimic tumor stiffness accurately.^[^ ^36^, ^37^ ^]^ The concentration ratio of components in hybrid hydrogels used for MCTS systems affects porosity and pore interconnectivity which are crucial for sufficient exchange of nutrients and waste products.^[^ ^38^, ^39^ ^]^

Natural hydrogels can be produced in a manner to replicate in vivo tumor microenvironments and improve the analysis of malignant behavior. **Table** **1** summarizes the advantages and disadvantages of protein, polysaccharide, and hybrid hydrogels. One of the main disadvantages is the batch‐to‐batch performance variations.^[^ ^40^, ^41^ ^]^ Therefore, if batch‐to‐batch variations are controlled by introducing technologies (e.g., microfluidics) that provide precise reagent sequence and distribution, reproducible and consistent results using biomimetic hydrogels can help minimize the animal models required for tumor studies and drug development. This could therefore advance the movement toward replacement, reduction and refinement in animal welfare and scientific research.^[^ ^42^ ^]^

**Table 1:** Advantages and disadvantages of protein, polysaccharide and hybrid hydrogels

Hydrogel	Advantages	Disadvantages	Ref.
Proteins	Natural origin Provide ligands recognized by cells Biocompatible Encourage cell proliferation. Physical crosslinking Ideal for cell dynamics and migration Angiogenesis promotion in MCTS systems	Batch‐to‐batch variations Low mechanical properties Contamination risk	^[^ ^15^, ^18^, ^19^, ^40^ ^]^
Polysaccharides	Natural origin Cell encapsulation appropriate Biodegradable Biocompatible, non‐toxic Easily functionalized Some are bioadhesive Structural support of cells Mild gelation conditions	Batch‐to‐batch variations Lack of cell adhesive ligands Low mechanical properties Might develop necrotic cores	^[^ ^24^, ^27^, ^28^, ^40^, ^41^ ^]^
Hybrids	Improve mechanical properties Improved pore interconnectivity Cell adhesion sites available Tunable characteristics Realistic recapitulation of tissue stiffness Good pore interconnectivity	Batch‐to‐batch variationsCrosslinking mechanisms may be difficult to achieve May be difficult to tune physicochemical characteristics	^[^ ^32^, ^33^, ^34^, ^36^, ^39^ ^]^

### 1.2 Cell Encapsulation Using Droplet‐Based Microfluidics

Multiphase droplet‐based microfluidics is a method extensively studied for cell encapsulation,^[^ ^43^ ^]^ as it is governed by the automated formation of droplets.^[^ ^44^ ^]^ To achieve droplet formation using microfluidics, immiscible phases (e.g., water, oil) and geometries, such as a T‐junction, flow‐focusing junction and co‐flow junctions, are frequently employed.^[^ ^45^, ^46^ ^]^ Droplet formation is governed by shear stresses and interfacial tension between the immiscible fluids and the channel walls, while the size of the droplets is determined by the flow rate ratio (FRR) and the size of the microfluidic channels.^[^ ^47^ ^]^ Single cell and multiple cell encapsulation protocols have been established using microfluidics and hydrogels, in order to investigate single cell behavior within a designated ECM, and to study cell‐cell and cell‐ECM interactions, respectively.^[^ ^48^, ^49^ ^]^ The application of microfluidics allows for experimental flexibility and agility as well as targeted and minimized resource consumption, which is of considerable benefit to cancer research, since the costs of drugs and reagents demand high precision.^[^ ^50^ ^]^

Entrapped cells in a confined environment, such as within hydrogel beads produced using microfluidic technology, can be analyzed on or off‐chip.^[^ ^51^ ^]^ Cell encapsulation within a microfluidic device can be achieved, usually, by combining droplet forming geometries and liquid polymers (as the dispersed phase), which, under crosslinking, form a biological scaffold for the cells to grow into 3D spheroids.^[^ ^45^, ^52^ ^]^ Relatively simple T‐junction microfluidic geometries have been used to enable cell encapsulation and cell growth assessment in the presence of hydrogel used as the ECM.^[^ ^53^ ^]^ However, more complex flow‐focusing junction geometries offer platforms that enable more sophisticated cell encapsulation procedures, control over gelation and protection of cells from potential harmful environments.^[^ ^54^, ^55^, ^56^ ^]^ Moreover, the density and viscosity of reagents flowing through microfluidic channels are important parameters that influence cell encapsulation. With appropriate microfluidic designs, the manipulation of these properties can enable different encapsulation structures, such as core‐shell hydrogel spatial arrangements.In addition to manipulating flow rates and reagents, microfluidics can integrate more complex fluidic circuitry and multi‐functionalism that is required for high throughput technologies in large scale cancer studies.^[^ ^7^, ^50^ ^]^ Finally, the combination of hydrogels and droplet microfluidics, offer the possibility to produce tumor tissue forming droplets and proceed to complex on or off chip drug assays, involving multiple cell types.

### 1.3 Cancer Spheroid Analysis and the Application of Droplet‐Based Microfluidics

Proteins,^[^ ^57^ ^]^ polysaccharides and hybrid hydrogels,^[^ ^58^, ^59^, ^60^ ^]^ have been produced using droplet microfluidic technologies for MCTS culture systems and drug evaluation. Flow focusing microfluidic platforms are capable to create various architectures, including core‐shell profiles (Figure 2b).^[^ ^61^, ^62^ ^]^ Agarwal et al. used droplet‐based microfluidics to form MCF‐7 microtumors in a collagen core surrounded by an alginate shell (Figure 2c).^[^ ^63^ ^]^ These microcapsules were formed at the junction of five microfluidic channels and when microtumors developed, they assessed anticancer drugs based on 3D vascularization experiments. Droplet‐based microfluidics, used for the formation of cell‐laden, hydrogel constructs, can enable diverse architectural formats, precision control over the manipulation of encapsulating materials and the process sequencing. This can enable multiple cell type co‐cultures in predefined microenvironments, that better simulate in vivo tissue complexity, thus making possible the development of high throughput platforms for drug treatments tailored to patients (Figure 2d).^[^ ^64^ ^]^

## 2 Drug Delivery Systems and Droplet‐Based Technology

Local delivery of therapeutic drugs can provide safe and effective ways for combating pathologies, such as cancer. Microparticles and liposomes can encapsulate drugs and deliver conjugates to the diseased tissue site, following passive and/or active release.^[^ ^65^, ^66^, ^67^ ^]^ The production of liposomes using traditional methods, include thin‐film hydration and ethanol injection,^[^ ^68^, ^69^ ^]^ and for microparticle production, membrane emulsification, precipitation and emulsion polymerization.^[^ ^70^ ^]^ Compared to conventional fabrication methods of microparticles and liposomes, microfluidic platforms offer benefits including reproducibility, control over size, precision processing and other as shown in **Table** **2**.

**Table 2:** Comparison of conventional and microfluidic production methods of microparticles and liposomes

Fabrication method	Advantages	Disadvantages	Ref.
Conventional	Mass production Simple and rapid	Broad size distributions Poor batch‐to‐batch reproducibility Post processing is required Larger volumes‐costly Difficult to control	^[^ ^65^, ^66^, ^67^ ^]^
Microfluidics	Reproducibility High throughput Narrow size distribution Control over size distribution Large scale manufacturing Precision processing Small volumes Flexibility in experimental design	Fabrication and microfluidic skills needed Low control over particle shape Low final concentrations in end product	^[^ ^71^, ^72^, ^73^, ^74^ ^]^

### 2.1 Microparticles as Drug Carriers

#### 2.1.1 Control of Content Release from Microparticles

**Figure 3:**
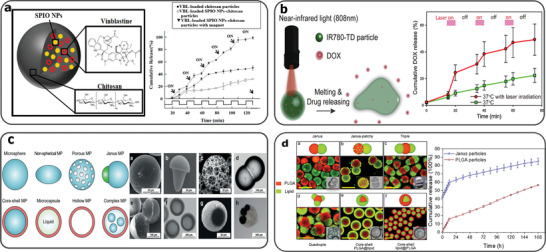
a) Magnetically responsive drug‐laden chitosan capsules fabricated using microfluidics. The chitosan droplets are loaded with superparamagnetic iron oxide nanoparticles (SPIO NPs) and chemotherapeutic drug, vinblastine (VBL), which is released by a pulsatile magnetic field. Reproduced with permission.^[^ ^88^ ^]^ Copyright 2019, MDPI. b) Droplet microfluidics fabricated near‐infrared (NIR) responsive 1‐tetradecanol microparticles, carrying doxorubicin (DOX)/ IR780 (photothermal agent). DOX release is achieved under NIR light pulses. Reproduced with permission.^[^ ^91^ ^]^ Copyright 2019, Elsevier. c) Graphic diagrams and microscopy images of possible polymer microparticles fabricated using microfluidics. Reproduced with permission.^[^ ^106^ ^]^ Copyright 2019, John Wiley and Sons. d) Lipid‐polymer Janus microparticles fabricated using microfluidics and solvent evaporation. Different structures achieved by altering the concentrations of the phases. a) Janus, b) Janus‐patchy, c) Triple, d) Quadruple, e,f) Core‐shell and sustained paclitaxel release from Janus microparticles. Reproduced with permission.^[^ ^113^ ^]^ Copyright 2019, Elsevier.

Microparticles can be used to encapsulate anticancer drugs^[^ ^75^ ^]^ and other molecules such as antibodies,^[^ ^76^ ^]^ peptides,^[^ ^77^ ^]^ enzymes,^[^ ^72^ ^]^ proteins,^[^ ^78^ ^]^ growth factors,^[^ ^79^ ^]^ indicators,^[^ ^80^ ^]^ and phages for different biomedical applications.^[^ ^81^ ^]^ These encapsulants can be released from the microparticles to the surroundings in either a passive or an active mechanism, depending upon the matrix material and the particle structure. The passive release mechanism is achieved through the degradation/hydrolysis of the matrix or the protective shell.^[^ ^82^ ^]^ Time‐dependent and sequential release of drugs can be realised by the design of multiple shells and multi‐core microparticle structures.^[^ ^83^, ^84^ ^]^ On the other hand, active release relies upon the microparticles being constructed from responsive materials.^[^ ^85^ ^]^ Such materials actuate a shape deformation that triggers a burst, or sustained release of encapsulants, responding to environmental variations and/or external stimuli.^[^ ^86^ ^]^ Stimuli‐responsive microparticles can be fabricated for the selective release of drugs, triggered by electrical fields,^[^ ^87^ ^]^ magnetic fields (Figure 3a),^[^ ^88^ ^]^ heating,^[^ ^89^ ^]^ ultrasound,^[^ ^90^ ^]^ infrared red light,^[^ ^91^ ^]^ pH change,^[^ ^92^ ^]^ redox,^[^ ^93^ ^]^ or the presence of enzymes.^[^ ^94^ ^]^ Microparticles can also be engineered to have multifunctionalities, such as sensing, directionality and mobility, which enables the delivery of encapsulants to the target area.^[^ ^95^, ^96^, ^97^ ^]^ 3.1.2. Matrix materials and structures of microfluidic formed microparticles

Using microfluidics, anticancer drug‐loaded microparticles can be prepared from a range of biocompatible materials, including lipid‐coated droplets,^[^ ^98^ ^]^ polymers,^[^ ^99^ ^]^ hydrogels,^[^ ^100^ ^]^ and metals.^[^ ^101^ ^]^ Among these, poly(lactic‐co‐glycolic acid) (PLGA), is a well‐researched polymeric material approved by the FDA as a safety drug vehicle.^[^ ^71^, ^75^, ^102^ ^]^ Biopolymers, such as proteins, gelatin, chitosan and alginate, are used to produce drug‐loaded microparticles using microfluidics to study cell responses, both in vitro and in vivo.^[^ ^103^, ^104^, ^105^, ^106^ ^]^ Other stimuli‐responsive microparticles produced using microfluidics include PEG,^[^ ^107^ ^]^ polylactide,^[^ ^108^ ^]^ graphene oxide,^[^ ^109^ ^]^ polyurea,^[^ ^110^ ^]^ polyester,^[^ ^93^ ^]^ poly(N‐vinylcaprolactam),^[^ ^89^ ^]^ and fatty alcohol (**Figure** **3**b),^[^ ^91^ ^]^ tailored with properties for various biomedical and drug delivery applications.

The morphologies of microparticles formed within microfluidic channel systems can be classified according to three categories, including i) spherical microbeads,^[^ ^111^ ^]^ ii) core‐shell shaped microcapsules,^[^ ^86^ ^]^ and iii) irregular shaped microparticles (Figure 3c). Irregular shaped microparticles, such as fibers, disks or anisotropic shapes, can be produced by alterations to the geometry constraints of fluidic channels, on emulsion droplets during their formation and consolidation processes.^[^ ^89^, ^112^ ^]^ Janus droplets have two or more distinct physical surface properties, and can be prepared either from the conjugation of several dispersed phases without mixing, or, from the phase separation of a droplet preloaded with mixed reagents (Figure 3d).^[^ ^113^ ^]^ These amphiphilic microparticles harness polarization from their structures, and can enable the staged release of drugs. Moreover, microfluidics can control the surface properties and porosity of microparticles, dependent upon the emulsion systems and the inflow parameters.^[^ ^114^, ^115^ ^]^ Such properties not only influence the release kinetics of the encapsulants from the microcapsules,^[^ ^116^ ^]^ but also affects the adhesion of microparticles to the in vivo environment.^[^ ^117^ ^]^

#### 2.1.2 Microfluidic Production of Microparticles for Drug Delivery and Screening

Microfluidics has been utilised for anticancer medicine development in the last few decades.^[^ ^118^, ^119^ ^]^ Microfluidic devices can be fabricated by subtractive micromachining processes, including reactive ion etching (RIE) on silicon,^[^ ^120^ ^]^ polymers and glasses, hot‐embossing,^[^ ^121^ ^]^ and injection molding of polymers,^[^ ^122^ ^]^ laser micromachining,^[^ ^123^ ^]^ and surface micromilling of metals, glasses, polymers and silicon,^[^ ^124^ ^]^ as well as more recently, additive manufacturing processes.^[^ ^125^ ^]^ In microfluidics, consistent droplet formation is essentially determined by continuous and stable fluid injection.^[^ ^126^ ^]^ which highly depends on the pumping system mechanisms.^[^ ^127^, ^128^, ^129^, ^130^ ^]^ With more recent progress in nanofluids, nano‐scale droplet forming junctions can be integrated within conventional microfluidic circuits to produce monodisperse nanoparticles for intracellular drug delivery and preclinical drug screening.^[^ ^131^ ^]^ The employment of droplet microfluidics for microparticle formation, also enables the integration with other drug delivery platforms, such as microneedles,^[^ ^132^ ^]^ acoustics,^[^ ^133^ ^]^ electrostatic atomization,^[^ ^134^ ^]^ electrospinning,^[^ ^135^ ^]^ magnetic‐assisted layer‐by‐layer coating to prepare and deliver drug loaded microparticles and scaffolds.^[^ ^136^ ^]^

With the development of new materials and microfluidic capabilities, sophisticated microparticles can be constructed from hybrid materials with hierarchical structures, that contributes to a programmable drug uptake, encapsulation and selective release process.^[^ ^137^ ^]^ Microfluidic processing methodologies are available for the mass fabrication of drug‐loaded microparticles, using parallel droplet forming arrays and multiplexed drug assays.^[^ ^138^ ^]^ These efforts are one step forward to manufacture smart drug microcarriers that harness on‐demand active targeting, and enable quantity‐controlled release of drugs for cancer therapy.

### 2.2 Liposomes as Drug Carriers

#### 2.2.1 Stimuli Responsive Liposomes

Artificial vesicles, usually termed liposomes, of both uni‐ and multi‐lamellar phospholipid bilayer structures around an aqueous core, can range in size from ∼30nm to 100s of µm.^[^ ^139^ ^]^ Such liposomal structures serve as very effective, “designer” drug delivery vehicles, due to their unique characteristics, including structural fluidity, biocompatibility, low toxicity and non‐immunogenicity making them ideal platforms for hydrophilic and hydrophobic drug encapsulation.^[^ ^140^, ^141^ ^]^ Drug‐loaded liposomes can be injected intravenously into animal models for drug related studies and their functionalisation may be beneficial for precision pharmacokinetics, while preventing adverse effects.^[^ ^142^ ^]^ Stealth‐liposomes (S‐liposomes), allow longer circulation times in the blood stream due to reduced uptake by reticuloendothelial system (RES),^[^ ^143^, ^144^ ^]^ as PEG makes liposomes more hydrophilic and prevent opsonins from adhering.^[^ ^145^ ^]^

A broadly used example of stimuli‐responsive liposomes are thermo‐sensitive liposomes (TSLs), which rely upon the phase transition of phospholipids, from gel to liquid.^[^ ^146^, ^147^ ^]^ Having a phase transition above physiological temperatures, liposomes become leaky and the contents leak out.^[^ ^148^ ^]^ The addition of lysolipids and polymers into liposomes reduces the transition temperature to that which is much closer to physiological temperatures.^[^ ^149^, ^150^, ^151^ ^]^

Some of the most known drug release external stimuli are summarized in **Table** **3**. Ultrasound is a widely available diagnostic imaging tool in clinical settings and can also serve as ideal means for triggering the selective release of drugs. Broadly, the application of ultrasound for liposomal drug release relies on i) thermally triggered release and, ii) non‐thermal membrane disruption.^[^ ^152^ ^]^ Other stimuli include high frequency and low frequency magnetic fields,^[^ ^153^, ^154^, ^155^ ^]^ NIR,^[^ ^156^, ^157^, ^158^ ^]^ and UV radiation.^[^ ^159^, ^160^ ^]^

**Table 3:** External stimuli and corresponding mechanism of drug release from liposomes

External stimulus	Mechanism of release	Ref.
Ultrasound High intensity focused ultrasound (HIFU) or Low frequency Ultrasound	• Ultrasonic beam focused at a focal point • Heating of diseased area administered with drug encapsulated liposomes • Microcavitation (bursting of gas bubbles, disruption of membrane leading to drug release)	^[^ ^152^, ^161^, ^162^ ^]^
Magnetic field High Frequency Magnetic fields or Low frequency alternating current (AC) magnetic fields	• Requires thermally responsive liposomes • Hyperthermia due to heating above the gel‐to‐liquid transition temperature • Magneto‐mechanical actuation of bilayer • Low frequency magnetic fields are considered safer	^[^ ^153^, ^154^, ^155^ ^]^
Near Infrared Light	• Photosensitive agent loaded liposomes. • Heating of particles encapsulated in liposomes upon radiation (magnetic and gold nanoparticles) • Heating effects causes microcavity and bilayer disruption • Other mechanism is cell penetrating peptide activation upon NIR light exposure	^[^ ^156^, ^157^, ^158^ ^]^
Ultraviolet Light	• UV light sensitive agents grafted onto liposomes • Activation of photosensitive agent upon exposure • Polymerization reactions between photosensitive lipid molecules	^[^ ^159^, ^160^ ^]^

Various stimuli‐responsive liposome constructs can release encapsulated content under external effects, whether they are composed of susceptible lipids, polymers, or other agents and particles. It is of considerable importance that biocompatible, drug‐laden, stimuli‐responsive liposomes, function appropriately, whilst simultaneously, ensuring that external stimuli do not induce undesired effects upon the host tissue. Drug delivery using liposomes can be tailored specifically to tumor types, and the physicochemical characteristics of the tumor microenvironment can be taken into advantage to trigger drug release by other internal and passive means.

#### 2.2.2 Tumor Targeted Liposomes

**Figure 4:**
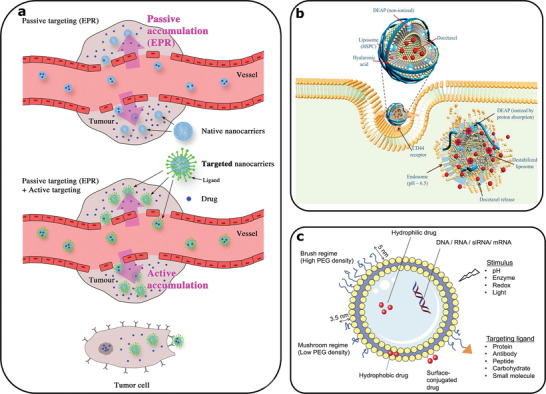
a) Representation of intravenous delivery of encapsulated drug in nanocarriers. (Top) Passive targeting of liposomal nanocarriers through the EPR effect, (Bottom) Ligand carrying liposomes for active tumor targeting. Reproduced with permission.^[^ ^9^ ^]^ Copyright 2019, John Wiley and Sons. b) An example of drug carrying tumor targeting liposome through a hyaluronic acid conjugate recognized by CD44 receptor. The liposome is endocytosed and acidic pH within a transformed cell causes the release of the docetaxel anticancer drug. Reproduced with permission.^[^ ^175^ ^]^ Copyright 2018, Elsevier. c) Presentation of multiple approaches to design stimuli responsive liposomes, while incorporating targeting ligands to achieve selective drug release. Reproduced with permission.^[^ ^180^ ^]^ Copyright 2017, John Wiley and Sons.

Tumor targeted liposomes follow either passive or active accumulation at cancerous tissues. The Enhanced Permeability and Retention (EPR) effect relates to drug nanocarriers, such as liposomes and microparticles, that passively meet and penetrate damaged vasculature and reach the location of the tumor.^[^ ^163^ ^]^ In addition to the passive targeting of tumors by the PEGylation of liposomes, the active targeting of liposomes has demonstrated the specific accumulation of liposomes at tumor sites (**Figure** **4**a).^[^ ^9^ ^]^ Antibodies,^[^ ^164^ ^]^ peptides,^[^ ^165^ ^]^ ssDNA,^[^ ^67^ ^]^ have all been used as ligands on liposomes, which are recognized by specific tumor, or endothelium receptors. It is believed that these methods for targeted drug delivery using Tumor Targeted Liposomes (TTLs) can resolve issues associated with safety and efficacy in cancer theranostics, while also reducing the suffering of pre‐clinical animal models.^[^ ^166^ ^]^

The tumor microenvironment differs from normal, healthy tissues, due to its altered characteristics, such as acidic pH, higher temperatures and hypoxia.^[^ ^167^ ^]^ Several researchers developed drug loaded ligand carrying liposomes that can endogenously release drug upon contact with such characteristics.^[^ ^167^, ^168^, ^169^ ^]^ Overexpressed cell surface receptors in tumors, are folate receptors,^[^ ^170^ ^]^ transferrin,^[^ ^171^ ^]^ epidermal growth factor receptors (EGFR),^[^ ^172^ ^]^ fibroblast growth factor receptors (FGFR),^[^ ^173^ ^]^ CD44 receptors (Figure 4b),^[^ ^174^, ^175^ ^]^ and the respective ligands grafted to liposomes can initiate endocytosis and drug release upon contact.^[^ ^176^ ^]^ Enzyme levels are also altered in the tumor microenvironment, and these have been utilised as an internal stimulus for drug release from liposomes, via liposome‐ tumor cell fusion.^[^ ^177^, ^178^ ^]^ Whilst the pH, temperature, and enzyme alterations at the tumor microenvironment act as initiators for the drug release from liposomes, internally induced release only, might not circumvent drug resistance.^[^ ^179^ ^]^

Externally induced drug release from liposomes includes light and heat producing methods, known as photodynamic therapy (PDT).^[^ ^180^ ^]^ PDT in combination with magnetic particles presents imaging applications.^[^ ^181^ ^]^ Tumor angiogenesis visualization using MRI imaging was achieved though the passive or active delivery of magnetic liposomes.^[^ ^181^, ^182^ ^]^ Li et al. also proposed a combined approach (PDT, MRI imaging and immunotherapy), using IR dye, MRI contrast agent, and anti‐EGFR antibody on liposomes for targeting, treating and imaging colorectal tumors.^[^ ^183^ ^]^ Figure 4c summarizes the possible modifications to produce functional and stimuli responsive liposomes, in order to encapsulate and deliver drugs effectively.^[^ ^180^ ^]^

#### 2.2.3 Microfluidics for Liposome Production and Drug Encapsulation

**Figure 5:**
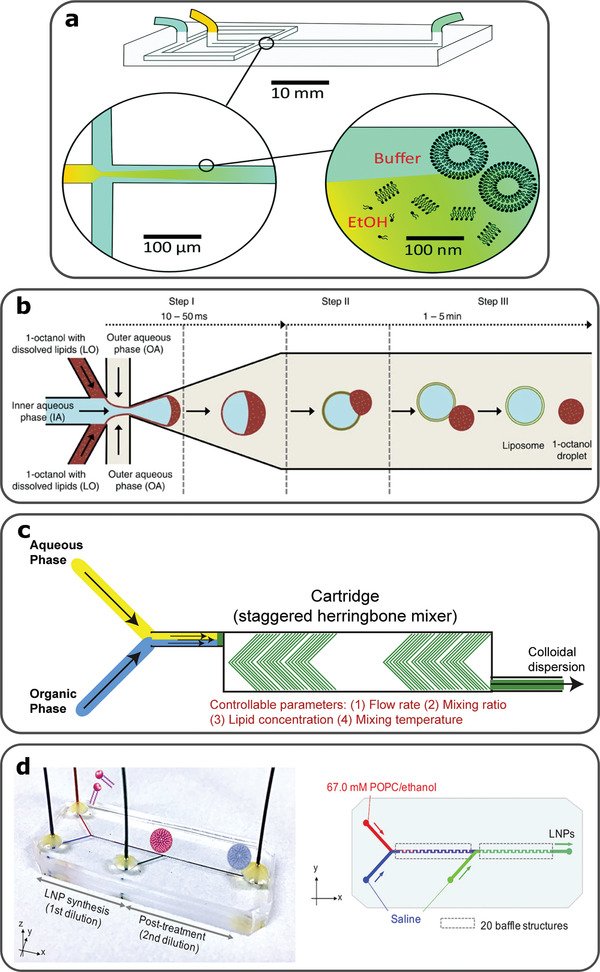
a) MHF device, with alcohol/lipids and water as inlets. Reciprocal diffusion at the interface of the two phases causes lipid hydration and vesicle formation, due to the self‐assembling nature of lipids. Reproduced with permission.^[^ ^185^ ^]^ Copyright 2021, Royal Society of Chemistry. b) Double emulsion of water in octanol/lipid and automated octanol extraction leads to formation of liposomes. Reproduced with permission.^[^ ^193^ ^]^ Copyright 2016, Nature Communications. c) Microfluidic device that combines a Y‐junction and SHM for liposome generation. Reproduced with permission.^[^ ^194^ ^]^Copyright 2019, Elsevier. d) Microfluidic device for liposome formation followed by post treatment using buffer for organic solvent removal. This microfluidic integrated post treating step avoids fusion of liposomes and does not affect encapsulation efficacy. Reproduced with permission.^[^ ^202^ ^]^ Copyright 2020, American Chemical Society.

Liposome formation is governed by the self‐assembling nature of lipids, due to their hydrophobic and hydrophilic parts, leading to the formation of vesicles. Microfluidic technologies have been utilized for liposome synthesis, by controlling the interface of flowing components, such as Microfluidic Hydrodynamic Focusing (MHF) devices.^[^ ^184^ ^]^ The process by which the liposomes self‐assemble in this MHF device is based on the reciprocal diffusion of alcohol/lipid and water at the downstream interface formed within the central channel of the chip, as shown in (**Figure** **5**a).^[^ ^185^ ^]^ Following studies determined the parameters of MHF devices that influence the diameter, dispersity and production rate of liposomes. Such parameters include the width of the alcohol stream,^[^ ^186^ ^]^ downstream turbulence,^[^ ^187^ ^]^ flow rate ratio,^[^ ^188^ ^]^ and width to height ratio of the microfluidic channels.^[^ ^189^, ^190^ ^]^ Increasing the number of diffusion interfaces within an MHF device increases the productivity of controlled sized liposomes, due to faster lipid hydration, as demonstrated using a double hydrodynamic focusing (DHF) microfluidic device.^[^ ^191^ ^]^ Cell‐sized vesicles have been also produced by double emulsions and organic solvent removal (Figure 5b).^[^ ^192^, ^193^ ^]^

Other microfluidic designs, apart from MHF devices, include the staggered herringbone micromixer (SHM), shown in Figure 5c.^[^ ^194^ ^]^ Microfluidic devices with integrated SHMs produce liposomes following chaotic advection and have been used for, the formation of stable SUVs encapsulating low water‐soluble drugs,^[^ ^195^, ^196^ ^]^ the production of liposomes for anticancer drug studies,^[^ ^197^ ^]^ as well as for the production of commercially available liposomes.^[^ ^198^ ^]^ Microfluidic geometries can host a sequence of reactions for liposome stabilization and control over drug encapsulation efficacy.^[^ ^199^, ^200^ ^]^ Microfluidic production of drug‐loaded liposomes using organic solvents require minimal post treatment to remove such solvents that influence the final product (Figure 5d).^[^ ^201^, ^202^ ^]^ Drug loaded liposomes produced using either MHF or SHM devices, have reached tumor sites due to their very narrow size and sufficient encapsulation efficacy.^[^ ^191^, ^200^, ^202^ ^]^ Mass production of these artificial vesicles by microfluidics is of great benefit to the pharmaceutical industry to produce amphipathic chemotherapeutic drugs, using considerably small volumes.

## 3 Artificial Cells as Programmable Drug Delivery Platforms

**Figure 6:**
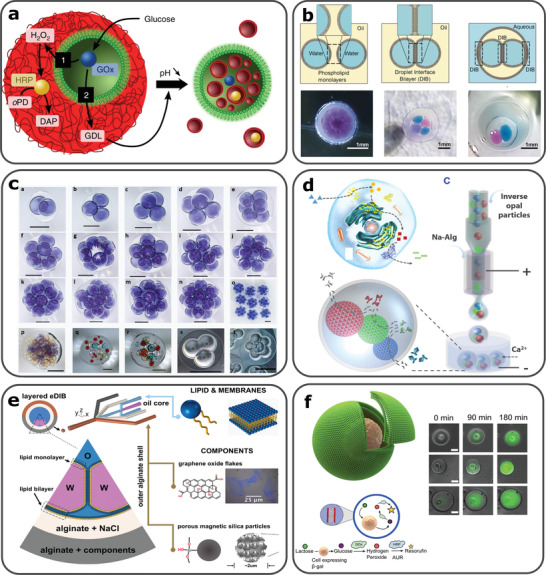
a) Guest‐host protocell construct for activation of synergistic or antagonistic behaviors. Glucose oxidase (GOx) containing proteinosome (guest protocell), trapped in a fatty acid micelle coacervate (host protocell). Depending on the glucose concentration of the surroundings, pathway 1 (synergistic) or 2 (antagonistic) is initiated. Low glucose concentration causes the coacervate to become fluorescent and high glucose concentration decreases pH and induces the formation of fatty acid vesicles. Reproduced with permission.^[^ ^211^ ^]^ Copyright 2018, Springer Nature. b) Schematic illustration for the formation of DIBs, multicore artificial cell constructs produced by droplet microfluidics and one or multiple DIBs and aqueous solutions encapsulated in a hydrogel capsule. Reproduced with permission.^[^ ^213^ ^]^ Copyright 2016, John Wiley and Sons. c) Highly compartmentalized capsules produced using novel bat‐wing junction, a–o) 2–15 water droplets encapsulated within solid semi‐permeable capsules, p–r) Different water droplets trapped in solid TMPTA solid capsules, s,t) encapsulated DIB networks in TMPTA/water/squalene. Reproduced with permission.^[^ ^240^ ^]^ Copyright 2017, Royal Society of Chemistry. d) Enzymatic reaction pathway in a natural eukaryotic cell and cell inspired hollow hydrogels encapsulating inverse opal particles with immobilized enzymes fabricated using microfluidics. Reproduced with permission.^[^ ^218^ ^]^ Copyright 2018, AAAS. e) Encapsulation of DIBs in alginate shells using non‐planar droplet microfluidic device. The outermost shell contains components such as magnetic particles, which offer mobility to the artificial cell construct in the presence of a magnet. Reproduced with permission.^[^ ^127^ ^]^. Copyright 2019, John Wiley and Sons. f) Vesicle‐cell engineered hybrid, where hydrolysis of lactose to glucose within the hybrid causes a chemical reaction that produces fluorescence. Reproduced with permission.^[^ ^230^ ^]^ Copyright 2018, Springer Nature.

In recent years, artificial cells have been developed based on compartmentalized particles and vesicles that have been applied to drug development and therapies.^[^ ^203^, ^204^ ^]^ One key aim of artificial cell research is to impart new functionalities upon either engineered natural cells through a top‐down approach, or through bottom‐up constructed protocells from non‐living elements, with de novo structure.^[^ ^205^ ^]^ Bottom‐up constructed artificial cells, also referred to as cell mimics,^[^ ^203^, ^206^ ^]^ may be imparted with one or more cell‐like features and behaviors, through the organization of biochemical reactions and the control of chemically‐mediated information, within internal compartmentalized structures, such as vesicles.^[^ ^207^, ^208^ ^]^ These structures can be formed by the self‐organization of molecules within an emulsion system, forming membrane‐bounded droplets with lipids, amphiphilic polymers and nanoparticles,^[^ ^209^ ^]^ as well as, membrane‐free systems from coacervation (**Figure** **6**a).^[^ ^210^, ^211^ ^]^

Several vesicle‐based protocell models have been designed, including liposomes, polymersomes, proteasomes, colloidosomes, and coacervates.^[^ ^212^ ^]^ Compartmentalized structures, including vesicles and droplet interface bilayers (DIBs) can be shaped within such models, as a counterpart to natural cellular organelles, and devised to orchestrate local chemical gradients and reactions (Figure 6b).^[^ ^213^, ^214^ ^]^ In comparison to other fabrication methods, droplet microfluidics has the advantages of precision control on the compartment size, structure and encapsulant (Figure 6c).^[^ ^215^, ^216^ ^]^ Thereby, the precision control afforded by the use of microfluidics, provides a versatile platform to develop new functionalities, such as membrane properties,^[^ ^217^ ^]^ sequential biochemical reactions (Figure 6d),^[^ ^218^ ^]^ cell‐free gene expression,^[^ ^219^ ^]^ and protein synthesis.^[^ ^220^ ^]^ In addition, an increasing number of different functionalities may be integrated within artificial cells as a simple program, involving sensing,^[^ ^221^ ^]^ predation,^[^ ^222^ ^]^ self‐sustainability,^[^ ^223^ ^]^ differentiation,^[^ ^224^ ^]^ and mobility (Figure 6e).^[^ ^127^ ^]^ This enables the exploration of the emergent, collective behavior of artificial cells to direct higher level activities, such as artificial cell‐based information integration and processing and chemical delivery decision making.^[^ ^225^ ^]^

With recent progress, artificial cells have been applied to various biotechnologies and drug development processes. Several works demonstrated the feasibility of employing artificial cells as smart carriers for anticancer drug delivery and screening applications.^[^ ^204^, ^226^, ^227^ ^]^ Encapsulated drug molecules may be released through certain drug metabolic pathways, while self‐reporting mechanisms may act as feedback. Such characteristics can be fundamentally enabled by the ability to control chemical information exchange during the interactions between artificial cells and living cells (Figure 6f). For example, artificial cells can be used to actuate or suppress the sensory pathways of microorganisms.^[^ ^228^ ^]^ To enable such efficient interactions, artificial cells and living cells, need to be appropriately integrated within the cell signaling range.^[^ ^229^ ^]^ This can be achieved by the encapsulation of living cells within an artificial cell chassis,^[^ ^230^ ^]^ or by spatial confinement using acoustic standing waves or fluidic structures.^[^ ^231^ ^]^ Living cells can be also bounded to artificial membranes via DNA tags.^[^ ^232^ ^]^ With the increasing precision of bioengineering, complex artificial cell colonies or prototissues can be constructed to facilitate artificial‐natural cell interactions, constructed through step‐by‐step emulsifications, or via droplet‐by‐droplet assembly with manual deposition, or 3D‐printing methods.^[^ ^233^ ^]^ These prototissue models can be programmed to have precise and functional geometries,^[^ ^234^ ^]^ and be responsive to external stimuli,^[^ ^235^, ^236^, ^237^, ^238^ ^]^ to trigger sequential biochemical reactions. Recently, pioneer studies demonstrate that prototissue models can be utilised to explore the metabolic pathway of natural cells and the development processes of organs.^[^ ^212^, ^239^ ^]^ With the development of artificial cellular constructs using bottom‐up or top‐down approaches, crucial information regarding cell growth, communication and interaction can be accumulated. Lastly, the researched literature indicates that artificial cells and their assembly could be a powerful platform to design novel drug metabolic pathways for programmable drug targeting and delivery.

## 4 Future Perspectives

**Figure 7:**
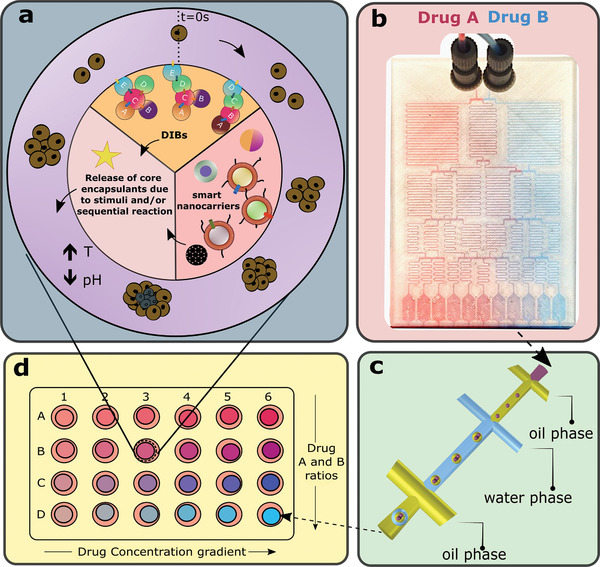
Schematic illustration of the stages required to perform anticancer drug screening using droplet incubators. a) A representation of a droplet incubator. The outer hydrogel shell of the droplet incubator hosts cancer cells and over incubation develop tumor spheres. The core is divided into three sections suggesting drug encapsulation and release systems. DIBs section proposes a cascade of reactions (A→E), that involves lipid bilayers and protein pores aiming the release of certain drug(s), to the shell. Another pie section describes possible smart nanocarriers (tumor targeting liposomes and microparticles) for the release of chemotherapeutic drugs under the influence of internal or external effects. Drug screening is possible by incorporating programmability within the context of artificial cells in the presence of tumorspheres. b) Hydrophilic Drug A and Drug B dilutions are performed within a microfluidic device. [Chip (shown) fabricated by 3D printing (unpublished data by the authors) for Worldcare Technologies Inc, using the design from,^[^ ^245^, ^246^ ^]^)] c) The candidate drug(s) flow into a device with droplet forming junctions which can generate triple emulsions (i.e., droplet incubator) at the outlet. d) Droplet incubators collected into a 24‐well plate for further analysis. Each well may host a single droplet incubator to generate a concentration gradient and drug ratio array, depending on the dilutions from the microfluidic chip in b).

We have shown that recent advances in precision droplet microfluidic technologies, is a key future enabler for both in vitro MCTSs formation, and anticancer drug delivery. Current anticancer drug screening employs a stepwise process, including (1) the development of cancer spheroid and organoid from individual cells, (2) the addition of candidate drugs with time points and concentration controls, and (3) the post processing and analyzing of samples. These discrete procedures normally require several sample transfers, continual manual handling, and the experimental results typically depend upon specific protocols and laboratory environments with inevitable operational errors. A future vision to enable a step change in anticancer drug development, involves the conjugation of both the cancer cells and the drug vehicle encapsulations (e.g., vesicles, microgels, polymeric capsules), within the same precision compartmentalized entity. Such a concept would comprise a novel microfluidically formed device, termed here as a “droplet incubator”, as a new platform for drug screening. The droplet incubator could incorporate natural cells, pharmaceuticals and other components, within separated intra‐capsule compartments, and enable the in vitro MCTS development and the interaction of micro tumors and drug candidates, in the same physical (incubator) construct, in a temporally‐programmed manner. The materials and the formation profiles of droplet incubators could provide the programmability to allow high throughput experiment, with parametric controls on the cell populations, drug quantification, and drug release and penetration. A schematic of such a droplet incubator is illustrated in **Figure** **7**. Tumor cell encapsulation and liposomal/microparticle/artificial cell, drug delivery vehicles, are combined within one precision‐assembled capsule, produced using multiphase droplet microfluidics. Such constructs would allow MCTS formation in a tissue mimicking environment, while drug release could be initiated in a programmable approach, involving the droplet structures, (bio)material properties, and external stimuli. The spatial arrangement of the drug carriers and tumor cells could be controlled with precision microfluidics, leading to various functional configurations.

The repeatable precision manipulation of small liquid quantities, through the use of multiphase microfluidics, is now enabling the physical formation of such programmable droplet incubators, incorporating spheroids and organoids, for advanced drug evaluation studies. Such droplet incubators can be incorporated within numerical models.^[^ ^241^, ^242^ ^]^ As an example, a numerical chemical compiler application is being developed, to enable the computer‐assisted design of microfluidics for the construction of artificial cells and droplet incubators.^[^ ^243^, ^244^ ^]^

## 5 Conclusion

Pathologies like cancer require patient specific treatments, due to the heterogenous genetic and epi‐genetic alterations across individuals, which cause limitations in the screening of anticancer drugs using conventional 2D cell cultures. To overcome some of these limitations, tumor growth and drug resistance can be modelled in vitro using 3D models, including MCTSs. Protein and polysaccharide polymers provide a wide range of materials that can be used to grow MCTSs in vitro, predict migration and angiogenesis, and program drug therapies to prevent aggressive tumor behavior. However, manual handling of these natural hydrogels may give rise to concentration variations, slower experimental procedures and suffer from human errors. Micro‐MCTSs grown in hydrogels that simulate animal tumor tissues can be produced using droplet microfluidics. Cell‐laden hydrogels using droplet forming geometries within a microfluidic device can be fabricated to obtain uniform, core‐shell, or multilayer structures. Controlled drug delivery to targeted tumor sites using microparticles and liposomes is an important advancement for avoiding adverse effects and reducing drug resistance. Targeting ligands for specific tumor cell receptors can be linked to microparticles and liposomes, and passively or actively accumulate at the tumor location and release the anticancer drug through internal or external stimuli. Microfluidic techniques provide the tools for the precision encapsulation of multiple drugs, molecules, and cells, and is enabling increasingly programmed reactions within a single physical construct (i.e., droplet incubator) for both tumor drug screening and targeted drug delivery.

## Conflict of Interest

The authors declare no conflict of interest.
